# Supplementary material for: Association between Sense of Loneliness and Quality of Life in Older Adults with Multimorbidity
Source: Int J Environ Res Public Health. 2023 Feb 1;20(3):2615. doi: 10.3390/ijerph20032615 (PMC9915419; doi:10.3390/ijerph20032615)
Supplement: Supplementary file 1 [file ijerph-20-02615-s001.zip › ijerph-2129524-supplementary.pdf]

The WHOQOL was dichotomize by no or few social relations and presence of good social relations. Pearson's coefficient was used to assess correlations between studied variables (Table S1).

Table S1. Pearson's R coefficients

	PWB	SWB	EWB	FWB	Multimorbidity	Sense of loneliness	WHOQOL AGE	ADL	IADL	GDS	CDQ
PWB	1	0.758**	0.829**	0.830**	-0.234**	-0.693**	0.741**	0.088	0.172*	-0.765**	-0.753**
SWB		1	0.746**	0.845**	-0.248**	-0.607**	0.618**	-0,018	0.103	-0.775**	-0.747**
EWB			1	0.821**	-0.200*	-0.734**	0.730**	-0,071	0.016	-0.814**	-0.772**
FWB				1	-0.269**	-0.695**	0.698**	0.032	0.234**	-0.779**	-0.789**
Multimorbidity					1	0.070	-0.232**	-0.069	-0.178*	0.191*	0.163*
Sense of loneliness						1	-0.677**	0.038	-0.034	0.679**	0.701**
WHOQOL AGE							1	0.106	0.144	-0.752**	-0.723**
ADL								1	0.560**	-0.040	-0.047
IADL									1	-0.067	-0.081
GDS										1	0.880**
CDQ											1

** p <= 0.01

* p <= 0.05

The multiple linear regression models was assessed to evaluate the associations between the dimensions of quality of life FACT_G and multimorbidity considering the effect of age, sex, sense of loneliness and WHOQOL **(Table3, S2, S3, S4).**

Table S2. Coefficients (dependent variable: SWB)

	B	p	95.0% CI	
			Lower	Upper
(Constant)	4.7	<0.001	2.895	6.506
MULTIMORBIDITY	-0.338	0.009	-0.589	-0.088
AGE	-0.022	0.051	-0.045	0
GENDER	-0.006	0.956	-0.238	0.225
SENSE OF LONELINESS	-0.401	<0.001	-0.55	-0.253
WHOQOL AGE	0.522	0.001	0.226	0.817

Table S3. Coefficients (dependent variable: EWB)

	B	p	95.0% CI	
			Lower	Upper
(Constant)	3.25	< 0.001	1.515	4.984
MULTIMORBIDITY	-0.063	0.604	-0.302	0.176
AGE	-0.001	0.939	-0.022	0.02
GENDER	-0.054	0.652	-0.292	0.183
SENSE OF LONELINESS	-0.494	< 0.001	-0.646	-0.343
WHOQOL AGE	0.885	< 0.001	0.587	1.182

Table S4. Coefficients (dependent variable: FWB)

	B	p	95.0% CI	
			Lower	Upper
(Constant)	4.556	< 0.001	3.072	6.04
MULTIMORBIDITY	-0.268	0.012	-0.475	-0.06
AGE	-0.023	0.013	-0.041	-0.005
GENDER	-0.001	0.991	-0.208	0.205
SENSE OF LONELINESS	-0.429	< 0.001	-0.562	-0.297
WHOQOL AGE	0.703	< 0.001	0.444	0.962
